# Estrogen Stimulates Proliferation and Differentiation of Neural Stem/Progenitor Cells through Different Signal Transduction Pathways

**DOI:** 10.3390/ijms11104114

**Published:** 2010-10-22

**Authors:** Makiko Okada, Akihisa Makino, Mitsunari Nakajima, Satoshi Okuyama, Shoei Furukawa, Yoshiko Furukawa

**Affiliations:** 1 Laboratory of Molecular Biology, Department of Biofunctional Analysis, Gifu Pharmaceutical University, Gifu, Japan; E-Mails: miyufreia@yahoo.co.jp (M.O.); makino-akihisa@jpo.go.jp (A.M.); furukawa@gifu-pu.ac.jp (S.F.); 2 Department of Pharmaceutical Pharmacology, Faculty of Pharmaceutical Sciences, Matsuyama University, Ehime, Japan; E-Mails: mnakajim@cc.matsuyama-u.ac.jp (M.N.); sokuyama@cc.matsuyama-u.ac.jp (S.O.)

**Keywords:** 17β-estradiol, bisphenol A, estrogen, neural stem cell, oligodendrocytes, proliferation, differeciation

## Abstract

Our previous study indicated that both 17β-estradiol (E2), known to be an endogenous estrogen, and bisphenol A (BPA), known to be a xenoestrogen, could positively influence the proliferation or differentiation of neural stem/progenitor cells (NS/PCs). The aim of the present study was to identify the signal transduction pathways for estrogenic activities promoting proliferation and differentiation of NS/PCs via well known nuclear estrogen receptors (ERs) or putative membrane-associated ERs. NS/PCs were cultured from the telencephalon of 15-day-old rat embryos. In order to confirm the involvement of nuclear ERs for estrogenic activities, their specific antagonist, ICI-182,780, was used. The presence of putative membrane-associated ER was functionally examined as to whether E2 can activate rapid intracellular signaling mechanism. In order to confirm the involvement of membrane-associated ERs for estrogenic activities, a cell-impermeable E2, bovine serum albumin-conjugated E2 (E2-BSA) was used. We showed that E2 could rapidly activate extracellular signal-regulated kinases 1/2 (ERK 1/2), which was not inhibited by ICI-182,780. ICI-182,780 abrogated the stimulatory effect of these estrogens (E2 and BPA) on the proliferation of NS/PCs, but not their effect on the differentiation of the NS/PCs into oligodendroglia. Furthermore, E2-BSA mimicked the activity of differentiation from NS/PCs into oligodendroglia, but not the activity of proliferation. Our study suggests that (1) the estrogen induced proliferation of NS/PCs is mediated via nuclear ERs; (2) the oligodendroglial generation from NS/PCs is likely to be stimulated via putative membrane-associated ERs.

## 1. Introduction

Embryonic neural stem cell (NSC)-transplantation or endogenous NSC-activation is a recent challenging approach for the treatment of neurological diseases or injuries to the central nervous system (CNS) [1–3]. NSCs comprise most of the progenitor cells (PCs) in the CNS [4,5] and are capable of self-renewal and multipotent differentiation into the three cell types of the CNS (neurons, astrocytes, and oligodendrocytes). The NSC-based therapies should be of note as the stem cell microenvironment or niche is important as various external signals have been shown to play a role in regulating their proliferation and differentiation [6]. Estrogens (female estrogenic hormones such as 17β-estradiol (E2) and estrogen-like endocrine-disrupting chemicals such as bisphenol A (BPA), *etc.*) are candidates for extrinsic regulators [7–10]. Our previous study showed that both E2 and BPA influence the fate of neural stem/progenitor cells (NS/PCs) when the cells are poorly supplied with mitogens or differentiation factors [10]. The aim of the present study was to investigate the underlying molecular mechanisms of estrogen actions on the proliferation and differentiation of NS/PCs.

Many estrogenic actions are known to be mediated via nuclear estrogen receptors (ERs), ERα and ERβ. Nuclear ERs function as ligand-activated transcription factors to regulate the expression of estrogen-responsive genes (ERE) [11]. In addition to nuclear ERs, membrane-associated ERs have recently been suggested to exist and to function, because some estrogenic effects occur within seconds to minutes, which is a finding that cannot be explained by a nuclear ER-mediated mechanism [12,13]. Investigations of nonclassical estrogen signaling suggest that ERα and ERβ, or ER-like proteins, are likely to be the membrane ERs in each target cells, and GPR-30, the orphan G protein-coupled receptors (GPCR)-like protein, was suggested to be one of the candidates of ER-like proteins [14,15]. Thus, to put it another way, the present study is aimed at identifying the signal transduction pathways for estrogenic activities promoting proliferation and differentiation of NS/PCs via well known nuclear ERs or putative membrane-associated ERs.

We have already confirmed that NS/PCs express both types of nuclear ERs [10], while even the existence of membrane-associated ERs has not yet been determined on NS/PCs. In various cells, the existence of membrane-associated ERs can be functionally confirmed [12,13]. Many reports have shown that E2 activates extracellular signal-regulated kinases (ERK)1/2, components of the mitogen-activated protein kinase (MAPK) signaling cascade, via membrane-associated ER, and that the activation of ERK1/2 is likely to be a key event to discriminate signal cascades dependent on ERs from those depending on non-nuclear ERs [16–19]. In the present study, we thus examined whether ERK 1/2-activation is shown in our NS/PCs after the treatment by estrogens in order to investigate the existence of membrane-associated ERs.

In order to confirm the participation of nuclear ERs on the estrogen actions, we investigated the protective efficacy of ICI-182,780 on the proliferation and differentiation of NS/PCs. ICI-182,780, a 7α–alkylsulfinyl analog of E2 [20], is able to bind to both ERα and ERβ with an affinity comparable to that of E2; and micromolar concentrations of this antagonist have been used to inhibit estrogen function [21]. In order to confirm the participation of putative membrane-associated ERs on the estrogen actions, we investigated the effect of a cell-impermeable E2, bovine serum albumin-conjugated E2 (E2-BSA) [17], on the proliferation or oligodendroglial differentiation of cultured NS/PCs.

## 2. Materials and Methods

### 2.1. Reagents

E2, BPA, and β-estradiol 6-(*O*-carboxymethyl)oxime: bovine serum albumin (E2-BSA), U0126 were purchased from Sigma (St. Louis, MO, USA). ICI-182,780 was obtained from Tocris (Ellisville, MO, USA). E2-BSA was separated from free E2 by use of a centrifugal filter, Microcon YM-30 (Millipore, Billerica, MA, USA). The E2-BSA used in this study was composed of BSA conjugated with 33 molecules of E2, *i.e.*, 3 × 10^−10^ M E2-BSA was equivalent to 10^−8^ M E2.

### 2.2. Primary Cultures

As previously described in detail [10], NS/PCs were prepared from the telencephalon of Wistar rats (Nippon SLC, Shizuoka, Japan) at E15, propagated by the neurosphere method, and then plated on poly-L-ornithine-coated plates or dishes for the experiments. The proliferation medium consisted of Dulbecco's modified Eagle's minimum essential medium nutrient mixture F-12 HAM (DMEM/F12) supplemented with insulin (25 μg/mL), apo-transferrin (100 μg/mL), progesterone (20 nM), putrescine (100 μM), sodium selenite (30 nM), penicillin (100 U/mL), and streptomycin (100 μg/mL). The required amount of FGF-2 (R&D Systems, Minneapolis, MN, USA) was added to the medium every day to achieve a final concentration of 10 ng/mL.

### 2.3. Western Immunoblot Analysis

Cells were plated on 6-well plates (1 × 10^5^ cells/cm^2^), and cultured for three days in the proliferation medium, for an additional day in FGF-2-free medium, then incubated with test drugs for various times, and the cell extracts were prepared as previously described [22]. The antibodies and their sources were the following: rabbit antibody against MAPK 1/2 (Erk1/2-CT) which recognizes the C-terminal 35 amino acids of the rat 44 kDa MAPK1/ERK1 and 42 kDa MAPK2/ERK2, from Upstate (Lake Placid, NY, USA); rabbit antibodies against phospho-p44/42 MAPK (Thr202/Tyr204) which recognizes the phosphorylated ERK1/2 (pERK1/2) from Cell Signaling (Danvers, MA, USA); and alkaline phosphatase-conjugated anti-rabbit IgG (H + L), from Promega (Madison, WI, USA).

### 2.4. BrdU Labeling

Cells were plated on micro slide/cover glasses (Matsunami Glass Ind. Ltd., Osaka, Japan) in 24-well plates (6 × 10^4^ cells/well) and cultured for three days in the proliferation medium, for an additional day in FGF-2-free medium containing the test compound, and then for 2 h in the presence of 10 μM 5-bromo-2′-deoxyuridine (BrdU; Sigma). The number of BrdU-positive cells was determined by an immunocytochemical technique using anti-BrdU mouse antibody (Sigma) and rhodamine-labeled anti-mouse IgG antibodies (Chemicon, Temecula, CA, USA), as described previously [10]. The total number of cells and the number of BrdU-positive cells (Figure 2A) were counted in seven arbitrarily selected fields of each well. For each treatment, 4–8 wells were analyzed; and the experiments were repeated more than three times. For statistical analysis Student’s *t-*test was used.

### 2.5. MTT Assay

As described previously [10], cells were plated (2 × 10^4^ cells/well) in 96-well plates and cultured for three days in the proliferation medium, for an additional day in FGF-2-free medium containing the test compound, and then for 4 h in the presence of 0.5 mg/mL of 3-[4,5-dimethyl-2-thiazolyl]-2,5-diphenyl tetrazolium bromide (MTT; Sigma). After the crystals had been dissolved in HCl/isopropanol, the absorbance at 570 nm was determined by using a micro plate reader (Bio-Rad, model 550). The cell number compared with that of the ethanol-treated control group was presented as a fold-increase. The total MTT converted to formazan by the cells (*i.e.*, total cell number) in all of the wells, had an absorbance that ranged from 0.2 to 0.75 at 570 nm.

### 2.6. Immunocytochemistry

Cells were plated on micro slide/cover glasses that had been placed in 24-well plates filled with the proliferation medium (4 × 10^4^ cells/well), and cultured for three days. They were then reacted for another five days with the test compound prepared in FGF-2-free medium. Immunocytochemical analysis was performed as described previously [10] using anti-nestin mouse antibody, anti-NG2 rabbit antibody (Chemicon), anti-βIII tubulin (Tuj-1) mouse antibody, anti-glial fibrillary acidic protein (GFAP) mouse antibody, anti-2′3′-cyclic nucleotide 3′-phosphodiesterase (CNPase) mouse antibody (Sigma), and rhodamine-labeled anti-mouse IgG antibody (Promega) or rhodamine-labeled anti-rabbit IgG antibodies (Chemicon). The total number of cells and the number of immunoreactive cells were counted in seven arbitrarily selected fields of each well. For each treatment, 4–8 different wells were analyzed, and the experiments were repeated more than three times. For statistical analysis, Student’s *t-*test was used.

## 3. Results and Discussion

### 3.1. Effect of Estrogens on the Phosphorylation of MAPK/ERK of NS/PCs

**Figure 1:**
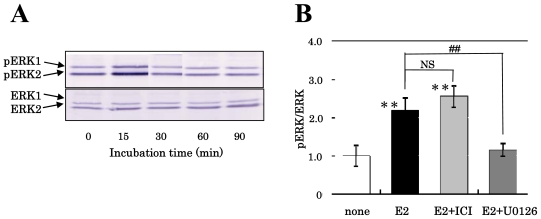
Effect of E2 on the phosphorylation of ERK of NS/PCs. (**A**) Cells were treated with 10^−8^ M E2 for the indicated times. The immunoreactive bands of pERK1/2 and ERK1/2 were scanned for intensity. (**B**) Cells were treated for 15 min with 10^−8^ M E2 after pretreatment for 1 h with 10^−6^ M ICI-182,780 or 30 min with 10^−5^ M U0126. The immunoreactive bands of pERK and ERK were scanned for intensity, and the ratios of pERK2/ERK2 are shown in the panel. Significant differences in values between the estrogen-treated and non-treated cells (**P < 0.01; Student’s *t* test) and in those indicated by the brackets are shown (^##^*P* < 0.01; Student’s *t* test, NS; not significant).

In order to functionally confirm the existence of membrane-associated ERs on NS/PCs, we assessed the effect of estrogens on the activation (phosphorylation) of ERK1/2 in the cultured NS/PCs. Our previous study showed that 2 ERK isoforms (ERK1 and ERK2) are present in NS/PCs [22]. As shown in Figure 1A, pERK1/2 increased transiently 15 min after the exposure of the cells to E2 at a concentration of 10^−8^ M, and declined to the control level 30 min after exposure, presenting the possibility that functional membrane-associated ERs were expressed on the NS/PCs and responded to E2 exposure.

When cells were pretreated with ICI-182,780, the activation of ERK2 induced by E2 was not blocked (Figure 1B) as previously indicated [23,24]. The reason why we analyzed the ratio of phosphorylated ERK2 (pERK2) per total ERK2 in Figure 1B is that only the ERK2 isoform has been suggested to be attributable to neural development [25] although ERK1 and ERK2 exhibit 84 % sequence homology and are coordinately activated through the MAPK cascade. The pretreatment with U0126, a selective inhibitor of MAPK kinase (MEK), inhibited E2-induced phosphorylation of ERK2 (Figure 1B), indicating the phosphorylation of ERK1/2 elicited by estrogen to be MEK dependent. These results suggested the possibility that our NS/PCs express membrane ERs.

### 3.2. Mechanism of Action of Estrogens on Proliferation of NS/PCs

**Figure 2:**
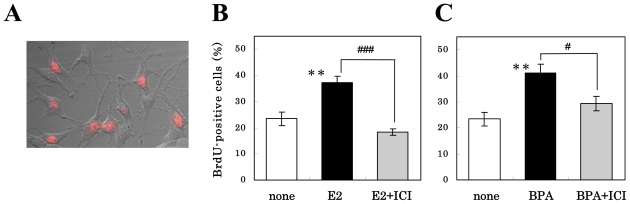
Effect of E2 or BPA, with or without ICI-182,780 pretreatment. on cell proliferation of NS/PCs, by measuring the incorporation of the thymidine analogue BrdU. The BrdU-positive cells were immunocytochemically examined (**A**), and their percentage in the presence of E2 (**B**) or BPA (**C**) with or without ICI-182,780 pretreatment is shown on the ordinate. The ratios of the value for the estrogen-treated cells to that value for the control cells (ethanol-treated cells) were calculated, and are shown on the ordinate. Values are presented as the mean ± SEM (n = 4–8, different cultures). Significant differences in values between the estrogen-treated and non-treated cells (**P < 0.01; Student’s *t* test) and in those indicated by the brackets are shown (^#^*P* < 0.05, ^###^*P* < 0.001; Student’s *t* test).

We previously showed that the administration of E2 or BPA to the NS/PCs stimulated their proliferation in the absence, but not in the presence, of FGF-2 [10]. In order to investigate whether the stimulatory effects of estrogens on the proliferation of NS/PCs were mediated through nuclear ERs, we pretreated cells for 1 h with 10^−6^ M ICI-182,780 before exposure to 10^−8^ M E2 or 10^−5^ M BPA for 24 h, and then determined the percentages of the cells in the S phase of the cell cycle by using the BrdU labeling method. As shown in Figure 2B and 2C, E2 and BPA, respectively, significantly increased the number of BrdU-positive cells compared with their number among the control cells; and pretreatment with ICI-182,780 significantly inhibited their effect. ICI-182,780 alone did not significantly decrease the BrdU-positive cells (not shown). These results suggest that the estrogens had the ability to stimulate proliferation of NS/PCs via nuclear ERs.

**Figure 3:**
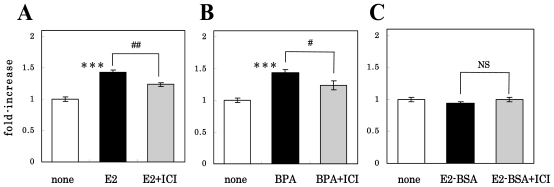
Effect of E2, BPA or E2-BSA, with or without ICI-182,780 pretreatment on cell proliferation of NS/PCs, by measuring the MTT assay. NS/PCs were pretreated or not with 10^−6^ M ICI-182,780 for 1 h, treated with E2 (**A**), BPA (**B**) or E2-BSA (**C**) for 24 h, and then subjected to the MTT assay. Values are presented as the mean ± SEM (n = 4–8, different cultures). Significant differences in values between the estrogen-treated and non-treated cells (***P < 0.001; Student’s *t* test) and in those indicated by the brackets are shown (^##^*P* < 0.01, ^###^*P* < 0.001; Student’s *t* test; NS, not significant).

We also checked the effect of the pretreatment with ICI-182,780 on the E2- or BPA-induced proliferation by use of the MTT assay. As shown in Figure 3A, compared with the MTT signals (indicating viable cells) from the non-treated control group, those from the E2-treated group were significantly greater; and pretreatment with ICI-182,780 significantly suppressed this effect. The cell number was unchanged when the cells were treated with ICI-182,780 alone (not shown). Figure 3B shows that similar results were obtained with BPA. These results confirmed those evaluated by the BrdU labeling experiment that the nuclear ERs participated in the stimulatory effect of estrogens on NS/PC proliferation.

In order to investigate the participation of putative membrane-associated ERs on the estrogen actions, we cultured NS/PCs for 24 h in FGF-2-free medium containing 3 × 10^−10^ M E2-BSA (equivalent to 10^−8^ M E2), a membrane-impermeable E2 conjugate, instead of E2. As shown in Figure 3C, E2-BSA did not increase the MTT signal intensity, thus suggesting that the membrane-associated ERs did not participate in the proliferation of NS/PCs. As expected, ICI-182,780 did not affect the cell number in the presence of E2-BSA.

Previous reports have shown the proliferative effect of E2 [7,8] and BPA [26] on cultured embryonic NSCs, though the action mechanisms involved in signal transduction were unknown. This is the first report suggesting a role for nuclear ERs in estrogen-induced proliferation of NS/PCs. It is now established that agonist-bound ERα has a strong positive effect on cell proliferation of several cell models including breast cancer cells [27]. Our NS/PCs expressed predominantly ERβ mRNA rather than the ERα mRNA that was predominant in neurons [10]. It will be necessary to clarify which ER has the most impact on proliferation of NS/PCs.

### 3.3. Mechanism of Action of Estrogens on Differentiation of NS/PCs

**Figure 4:**
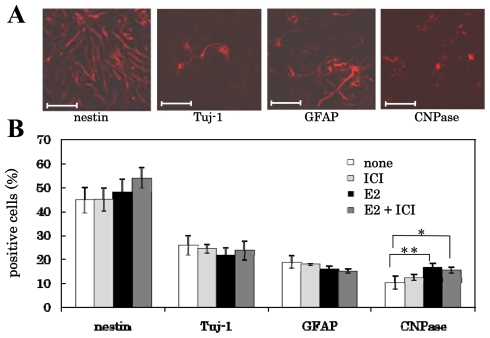
Effect of E2-treatment and/or ICI-182,780-pretreatment on the differentiation of NS/PCs. The cells were examined by immunocytochemistry using anti-nestin, anti-Tuj-1, anti-GFAP or anti-CNPase antibodies (Scale bar = 50 μm) (**A**). The percentage of cells positive for each marker was calculated, and is shown on the ordinate (**B**). Their values were expressed as the mean ± SEM (n = 4–16). Significance, *p < 0.05, **p < 0.01 *versus* control; Student’s *t* test.

We previously showed that E2- or BPA-treatment increased the ratio of the oligodendrocytes generated from the NS/PCs to total cells; however, this ratio did not change when the cells were stimulated with platelet-derived growth factor (PDGF) or with neurotrophin-3 [10]. We tested the action mechanisms of these estrogens on the differentiation of NS/PCs into oligodendroglia. NS/PCs were pretreated for 1 h with or without 10^−6^ M ICI-182,780, and cultured for five days in FGF-2-free medium containing 10^−8^ M E2. Figure 4 shows that the proportion of nestin-positive (immature neural stem cells), Tuj-1-positive (neurons) and GFAP-positive (astrocytes) cells did not change significantly, but the percentage of CNPase-positive (oligodendrocytes) cells was significantly increased by E2-treatment as previously shown [10]; and that the pretreatment of the cells with ICI-182,780, a classical ER antagonist, did not affect the percentage of these positive cells.

**Figure 5:**
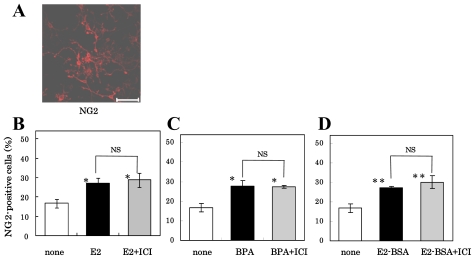
Effect of E2, BPA, or E2-BSA with or without ICI-182,780-pretreatment on the oligodendroglial differentiation of NS/PCs. The cells were examined by immunocytochemistry using anti-NG2 antibodies (Scale bar = 50 μm) (**A**), and NG2-positive cells in the various cultures was determined. Their values in the presence of E2 (**B**), BPA (**C**) or E2-BSA (**D**) are expressed as the mean ± SEM (n = 4–16) on the ordinate. Significance, *p < 0.05, **p < 0.01 *versus* control; Student’s *t* test. NS, not significant.

We then pretreated NS/PCs for 1 h with or without 10^−6^ M ICI-182,780 and cultured for five days in FGF-2-free medium containing 10^−8^ M E2, 10^−5^ M BPA or 3 × 10^−10^ M E2-BSA. As shown in Figure 5, the percentage of NG2-positive oligodendrocyte precursor cells in the NS/PC cultures was significantly increased by BPA or E2-BSA as well as E2 (closed bar) compared with the percentage in control cultures (open bar), and the pretreatment of the cells with ICI-182,780 did not reverse the differentiation-inducing effect of E2, BPA or E2-BSA (shaded bar). ICI-182,780 alone did not affect the percentage of the NG-2-positive cells (data not shown).

These results suggested that estrogen-induced glial generation from NS/PCs was mediated, not through nuclear ERs, but probably through membrane-associated ERs. Recently evidence was presented that STX, a synthetic selective ER modulator, and G1, the GPR-30 agonist, are beneficial tools as non-classical ER agonist [28]. Both STX and G1 do not bind to or activate the nuclear ERs, but activate MAPK pathway. It will be necessary to investigate the effect of these compounds on NS/PCs in near future.

## 4. Conclusions

This study demonstrates that the estrogen-induced proliferation of NS/PCs was mediated via nuclear ERs, and suggests that the oligodendroglial generation from NS/PCs was stimulated by putative membrane-associated ERs.

## References

[1] Ebert AD Beres AJ Barber AE Svendsen CN Human neural progenitor cells over-expressing IGF-1 protect dopamine neurons and restore function in a rat model of Parkinson's disease Exp. Neurol 2008 209 213 223 18061591 10.1016/j.expneurol.2007.09.022

[2] Imitola J Khoury SJ Neural stem cells and the future treatment of neurological diseases: Raising the standard Methods Mol. Biol 2008 438 9 16 18369745 10.1007/978-1-59745-133-8_2

[3] Lu P Jones LL Snyder EY Tuszynski MH Neural stem cells constitutively secrete neurotrophic factors and promote extensive host axonal growth after spinal cord injury Exp. Neurol 2003 181 115 129 12781986 10.1016/s0014-4886(03)00037-2

[4] Lovell-Badge R The future for stem cell research Nature 2001 414 88 91 11689952 10.1038/35102150

[5] Song RX Santen RJ Apoptotic action of estrogen Apoptosis 2003 8 55 60 12510152 10.1023/a:1021649019025

[6] Watt FM Hogan BLM Out of eden: Stem cells and their niches Science 2000 287 1427 1430 10688781 10.1126/science.287.5457.1427

[7] Brännvall K Korhonen L Lindholm D Estrogen-receptor-dependent regulation of neural stem cell proliferation and differentiation Mol. Cell. Neurosci 2002 21 512 520 12498791 10.1006/mcne.2002.1194

[8] Brännvall K Bogdanovic N Korhonen L Lindholm D 19-Nortestosterone influences neural stem cell proliferation and neurogenesis in the rat brain Eur. J. Neurosci 2005 21 871 878 15787693 10.1111/j.1460-9568.2005.03942.x

[9] McEven BS Estrogen actions throughout the brain Recent Prog. Horm. Res 2002 57 357 384 12017552 10.1210/rp.57.1.357

[10] Okada M Murase K Makino A Nakajima M Kaku T Furukawa S Furukawa Y Effects of estrogens on proliferation and differentiation of neural stem/progenitor cells Biomed. Res 2008 29 163 170 18614850 10.2220/biomedres.29.163

[11] Beato M Herrlich P Schutz G Steroid hormone receptors: Many actors in search of a plot Cell 1995 83 851 857 8521509 10.1016/0092-8674(95)90201-5

[12] Falkenstein E Tillmann HC Christ M Feuring M Wehling M Multiple actions of steroid hormones: A focus on rapid, nongenomic effects Pharmacol. Rev 2000 52 513 556 11121509

[13] Toran-Allerand CD Minireview: A plethora of estrogen receptors in the brain: Where will it end? Endocrinology 2004 145 1069 1074 14670986 10.1210/en.2003-1462

[14] Qiu J Bosch MA Tobias SC Krust A Graham SM Murphy SJ Korach KS Chambon P Scanlan TS R⊘nnekleiv OK Kelly MJ A G-protein-coupled estrogen receptor is involved in hypothalamic control of energy homeostasis J. Neurosci 2006 26 5649 5655 16723521 10.1523/JNEUROSCI.0327-06.2006 PMC2678732

[15] Thomas P Pang Y Filardo EJ Dong J Identity of an estrogen membrane receptors coupled to a G protein in human breast cancer cells Endocrinology 2005 146 624 632 15539556 10.1210/en.2004-1064

[16] Björnström L Sjöberg M Mechanism of estrogen receptor signaling convergence of genomic and nongenomic actions on target genes Mol. Endocrinol 2005 19 833 842 15695368 10.1210/me.2004-0486

[17] Bouskine A Nebout M Mograbi B Brücker-Davis F Roger C Fenichel P Estrogens promote human testicular germ cell cancer through a membrane-mediated activation of extracellular regulated kinase and protein kinase A Endocrinology 2008 149 565 573 18039775 10.1210/en.2007-1318

[18] Titolo D Mayer CM Dhillon SS Cai F Belsham DD Estrogen facilitates both phosphatidylinositol 3-kinase/Akt and ERK1/2 mitogen-activated protein kinase membrane signaling required for long-term neuropeptide Y transcriptional regulation in clonal, immortalized neurons J. Neuroci 2008 28 6473 6482 10.1523/JNEUROSCI.0514-08.2008 PMC6670897 18562618

[19] Mendelsohn EM Karas RH Rapid progress for non-nuclear estrogen receptor signaling J. Clin. Inv 2010 120 2277 2279 10.1172/JCI43756 PMC2898619 20577045

[20] Wakeling AE Dukes M Bowler J A potent specific pure antiestrogen with clinical potential Cancer Res 1991 51 3867 3873 1855205

[21] Howell A DeFriend DJ Robertson JF Blamey RW Anderson L Anderson E Sutcliffe FA Walton P Pharmacokinetics, pharmacological and anti-tumour effects of the specific anti-oestrogen ICI 182,780 in women with advanced breast cancer Br. J. Cancer 1996 74 300 308 8688341 10.1038/bjc.1996.357 PMC2074590

[22] Furukawa Y Urano T Minamimura M Nakajima M Okuyama S Furukawa S 4-Methylcatechol-induced heme oxygenase-1 exerts a protective effect against oxidative stress in cultured neural stem/progenitor cells via PI3 kinase/Akt pathway Biomed. Res 2010 31 45 52 20203419 10.2220/biomedres.31.45

[23] Dominguez R Jalali C de Lacalle S Morphological effects of estrogen on cholinergic neurons *in vitro* involves activation of extracellular signal-regulated kinases J. Neurosci 2004 24 982 990 14749443 10.1523/JNEUROSCI.2586-03.2004 PMC3182120

[24] Zhao L O’Neill K Brinton RD Estrogenic agonist activity of ICI 182,780 (faslodex) in hippocampal neurons: Implications for basic science understanding of estrogen signaling and development of estrogen modulators with a dual therapeutic profile J. Pharm. Exp. Ther 2006 319 1124 1132 10.1124/jpet.106.109504 16951259

[25] Samuels IS Karlo JC Faruzzi AN Pickering K Herrup K Sweatt JD Saitta SC Landreth GE Deletion of ERK2 mitogen-activated protein kinase identifies its key roles in cortical neurogenesis and cognitive function J. Neurosci 2008 28 6983 6995 18596172 10.1523/JNEUROSCI.0679-08.2008 PMC4364995

[26] Kudo C Wada K Masuda T Yonemura T Shibuya A Fujimoto Y Nakajima A Niwa H Kamisaki Y Nonylphenol induces the death of neural stem cells due to activation of the caspase cascade and regulation of the cell cycle J. Neurochem 2003 88 1416 1423 15009642 10.1046/j.1471-4159.2003.02270.x

[27] Song RX Santen RJ Apoptotic action of estrogen Apoptosis 2003 8 55 60 12510152 10.1023/a:1021649019025

[28] Lebesque D Traub M Butte-Smith MD Chen C Zukin RS Kelly MJ Etgen AM Acute administration of non-classical estrogen receptor agonists attenuates ischemia-induced hippocampal neuron loss in middle-aged female rats PLoS One 2010 30 12950 12957 10.1371/journal.pone.0008642 PMC2799530 20062809

